# Reversal of Pacing‐Induced Cardiomyopathy After Upgrade to Left Bundle Branch Area Pacing: A Case Report

**DOI:** 10.1002/ccr3.72919

**Published:** 2026-06-17

**Authors:** Mengqi Yeh, Shunsong Chen, Shanshan He, Ke Liu, Kai Yang, Jiang Wang, Zhiying Zhao, Feiyan Shang, Jinrui Guo

**Affiliations:** ^1^ Fuwai Yunnan Hospital, Chinese Academy of Medical Sciences, Affiliated Cardiovascular Hospital of Kunming Medical University Kunming China

**Keywords:** left bundle branch area pacing, pacing‐induced cardiomyopathy, permanent pacemaker implantation, right ventricular pacing

## Abstract

High‐burden right ventricular pacing induced cardiomyopathy in a 47‐year‐old male; upgrading to left bundle branch area pacing may lead to rapid and sustained recovery of cardiac function.

## 1 Introduction

A significant drawback of traditional right ventricular pacing is that approximately 20%–30% of patients may develop ventricular dysfunction, a condition termed pacing‐induced cardiomyopathy (PICM) [1]. Historically, the primary treatment involved upgrading to cardiac resynchronization therapy (CRT) via biventricular pacing. With the advent of left bundle branch area pacing (LBBAP), it emerges as a promising alternative for managing PICM. We present a case of PICM successfully treated with LBBAP.

While the efficacy of LBBAP in reversing PICM has been increasingly documented, the present case exhibits several unique and instructive features that merit particular attention. First, the patient represents a relatively young cohort (47 years old) with a complex anatomical substrate, having undergone ventricular septal defect closure with an occluder device 14 years prior. The presence of an occluder within the ventricular septum presents a distinct technical challenge and raises theoretical concerns regarding septal perforation and lead stability during LBBAP lead deployment. This case underscores that LBBAP can be safely and effectively executed even in the presence of intracardiac hardware, a scenario infrequently reported in the literature. Second, this case highlights a remarkably rapid and complete reverse remodeling following the upgrade to physiological pacing. Within just 2 months, the left ventricular ejection fraction (LVEF) improved from a severely depressed 35% to 45%, accompanied by a significant reduction in left ventricular end‐diastolic diameter (LVEDD) from 65 to 50 mm, and normalization of NT‐proBNP levels. This swift recovery contrasts with the often slower and sometimes incomplete response observed with traditional BiVP‐CRT upgrades, particularly in patients with long‐standing dyssynchrony [2].

Furthermore, this report adds valuable long‐term follow‐up data to the growing body of evidence supporting LBBAP as a superior alternative to BiVP for PICM. While previous studies have established the acute and short‐term hemodynamic benefits of LBBAP, the sustained improvement in echocardiographic parameters and NYHA functional class observed at the 1‐year mark in this patient reinforces the durability of the response. Given the technical difficulties encountered during the procedure—including subclavian vein stenosis and adhesion precluding ipsilateral lead replacement—the successful contralateral LBBAP implant demonstrates the versatility of this approach in managing challenging lead revision [3]. Therefore, this case provides a compelling narrative for clinicians considering upgrading strategies in young, high‐burden RVP patients with concomitant structural heart disease, offering a concrete example of how LBBAP can overcome anatomical hurdles to achieve optimal physiological resynchronization.

We present a case of PICM successfully treated with LBBAP.

## 2 Case Presentation

A 47‐year‐old male was admitted with “progressive exertional dyspnea over one month, occurring five years after permanent pacemaker implantation.” Five years prior (May 9, 2019), electrocardiography had revealed third‐degree atrioventricular block. A permanent dual‐chamber pacemaker was implanted via the left subclavian vein, with the ventricular lead positioned at the right ventricular septum. In August 2021, the patient developed a pacemaker pocket infection, complicated by skin ulceration. Management involved complete lead extraction and surgical debridement, followed by implantation of a new dual‐chamber pacemaker via the right subclavian vein, again with septal positioning of the ventricular lead (Figure 1A). Echocardiography performed on August 26, 2021, showed a LVEDD of 51 mm, a LVEF of 66%, and an NT‐proBNP level of 37.75 pg/mL. A follow‐up echocardiogram on August 8, 2023, revealed a stable LVEDD of 51 mm, but the LVEF had decreased to 58%. One month prior to the current admission, the patient reported exertional dyspnea without lower limb edema. His medical history included ventricular septal defect closure with an occluder device 14 years earlier, in 2007. Physical examination was unremarkable.

**FIGURE 1:**
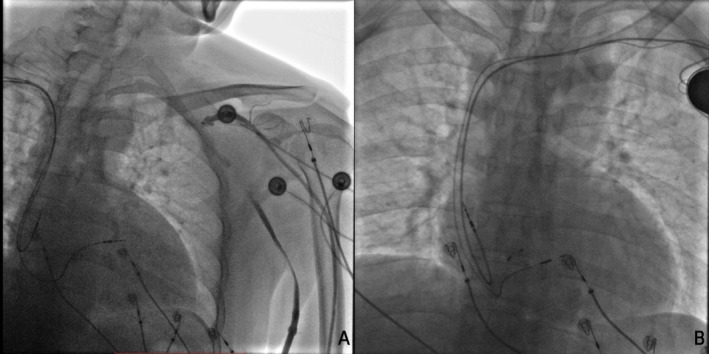
Preoperative and postoperative fluoroscopic images. (A) Preoperative image showing right ventricular septal pacing. (B) Postoperative image showing left bundle branch area pacing.

Findings upon current admission were as follows: Electrocardiography (Figure 2A) showed: (1) Sinus rhythm; (2) Atrial sensing with ventricular pacing; and (3) Normal pacemaker function, with a paced QRS duration of 182 ms. Echocardiography (Figure 3A,B) revealed a LVEDD of 65 mm, a left ventricular end‐diastolic volume (LVEDV) of 259 mL, a left ventricular end‐systolic volume LVESV of 169 mL, a LVEF of 35%, diffuse left ventricular hypokinesis, and an NT‐proBNP level of 922.8 pg/mL. Pacemaker interrogation indicated a ventricular pacing proportion exceeding 99.9%.

**FIGURE 2:**
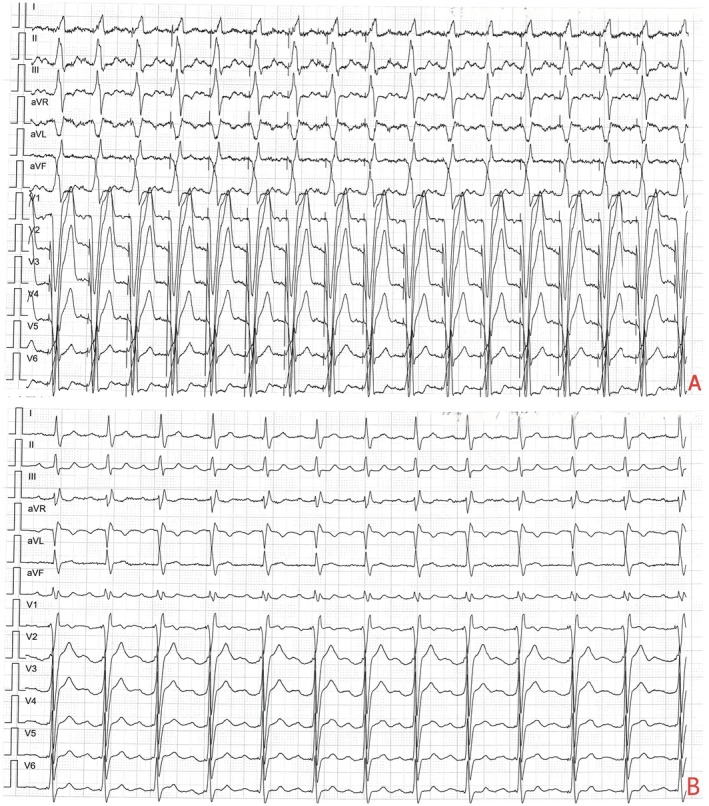
Preoperative and postoperative electrocardiograms. (A) Preoperative ECG demonstrating right ventricular septal pacing with a QRS duration of 182 ms. (B) Postoperative ECG demonstrating left bundle branch area pacing with a QRS duration of 136 ms.

**FIGURE 3:**
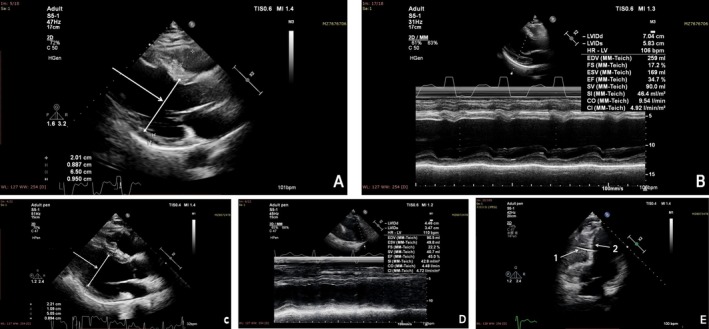
Preoperative and postoperative echocardiographic images. (A) Preoperative parasternal long‐axis view showing a left ventricular end‐diastolic diameter of 65 mm (arrow). (B) Preoperative M‐mode measurement showing a left ventricular ejection fraction of 35%. (C) Two‐month postoperative parasternal long‐axis view showing a left ventricular end‐diastolic diameter of 50 mm (arrow). (D) Two‐month postoperative M‐mode measurement showing an ejection fraction of 45%. (E) Two‐month postoperative echocardiogram showing the ventricular lead traversing the interventricular septum and pacing the left ventricular septal surface (1, electrode; 2, ventricular septum).

Exclusion of alternative etiologies: Coronary artery disease was considered unlikely given the absence of cardiovascular risk factors (no hypertension, diabetes, dyslipidemia, or smoking), no exertional angina, normal troponin levels, and no ischemic ECG changes. Myocarditis was excluded by the absence of prodromal illness, fever, elevated inflammatory markers (CRP, ESR), or regional wall motion abnormalities on echocardiography. Idiopathic dilated cardiomyopathy (DCM), a key differential diagnosis, was effectively ruled out by the absence of any family history of cardiomyopathy or sudden cardiac death, the clear temporal relationship between the onset of left ventricular dysfunction and prolonged high‐burden right ventricular pacing (> 99%), and, most definitively, the dramatic and complete reversal of ventricular function and dimensions (LVEF from 35% to 52%, LVEDD from 65 to 48 mm) within 1 year of upgrading to physiological pacing—a recovery trajectory characteristic of PICM and atypical for idiopathic DCM. Infiltrative cardiomyopathy (e.g., amyloidosis, sarcoidosis) had no clinical stigmata (no neuropathy, skin changes, renal dysfunction, or arrhythmias suggestive of infiltration). Importantly, the patient's implanted dual‐chamber pacemaker was not MRI‐conditional (non‐MRI compatible). Therefore, cardiac magnetic resonance imaging was contraindicated and could not be performed. The lack of CMR is acknowledged as a limitation, but the clinical picture—> 99% RV pacing burden, LVEF decline from 66% to 35%, and dramatic recovery after LBBAP—is highly consistent with PICM.

## 3 Methods

### 3.1 Differential Diagnosis

Considering the patient's 5‐year history of pacemaker implantation, the decline in LVEF from 66% to 35%, the absence of exertional angina, no history of smoking, alcoholism, hypertension, or diabetes, and a right ventricular septal pacing proportion of over 99%, the patient was diagnosed with: Sinus rhythm with third‐degree atrioventricular block, Status post permanent dual‐chamber pacemaker implantation, PICM, NYHA class II heart failure, and Status post ventricular septal defect closure.

According to the diagnostic criteria for PICM (baseline LVEF > 50%, absolute decline ≥ 10% to a final LVEF < 50%, high RV pacing burden, and exclusion of other causes), the patient met all criteria for this diagnosis [4].

### 3.2 Investigations

Diagnostic investigations included serial electrocardiography, transthoracic echocardiography, and NT‐proBNP measurement. Pacemaker interrogation confirmed > 99% ventricular pacing burden.

### 3.3 Treatment

Patient's existing pacemaker generator (right‐sided) had sufficient remaining battery life and no evidence of infection. Therefore, a system revision was performed on September 27, 2024. The right subclavian vein approach was abandoned due to severe stenosis and adhesions from the previous lead; thus, a left subclavian puncture was performed. A new atrial lead (Medtronic 5072‐52) was placed in the right atrial appendage. A 3830 electrode (Medtronic) was then advanced via a C315‐His sheath, and left bundle branch area pacing (LBBAP) was performed. The existing generator was disconnected from the original right‐sided leads (which were later removed by manual traction), connected to the new left‐sided leads, and repositioned in a new left pectoral pocket (Figure 1B). No left ventricular (coronary sinus) lead was implanted; hence, this upgrade was to LBBAP alone, not conventional biventricular pacing.

Procedural details: Fluoroscopy time was 4.9 min. Immediate post‐implant pacing threshold for the LBBAP lead was 0.5 V at 0.5 ms, impedance 421 Ω (Table 1). Lead depth in the interventricular septum was 10.5 mm from the right septal surface. A final intraseptal depth of 10.5 mm was necessary for stable left bundle capture and was well tolerated without septal perforation as confirmed by echocardiography. Left bundle capture was confirmed by (1) paced RBBB morphology (rsR′ in V1, qR in V6); (2) stimulus‐to‐LV activation time (stim‐LVAT) of 72 ms; and (3) transition from nonselective to selective capture at 0.8 V at 0.5 ms, with a discrete left bundle potential. Postoperative ECG showed a paced QRS duration of 136 ms (Figure 2B), notably shorter than pre‐procedural right ventricular septal pacing. The procedural details and left bundle capture criteria are summarized in Table 2.

**TABLE 1:** Pacing and sensing parameters during implantation and at the two‐month follow‐up.

Parameter	Atrium	Ventricle
**During implantation**		
Threshold (V)	0.5 at 0.5 ms	0.5 at 0.5 ms
Impedance (Ω)	565	421
Sensing (mV)	5.6	N/A
**Two‐month follow‐up**		
Threshold (V)	0.7 at 0.5 ms	0.8 at 0.5 ms
Impedance (Ω)	730	890
Sensing (mV)	3.0	N/A
Ventricular pacing (%)	–—	99

*Note:* All threshold measurements were performed at a pulse width of 0.5 ms. Ventricular sensing values were not measured (N/A) at both time points.

Abbreviation: N/A, not available.

**TABLE 2:** Procedural details and LBBAP capture criteria.

Parameter	Value
Fluoroscopy time	4.9 min
Immediate LBBAP threshold	0.5 V at 0.5 ms
Lead depth in septum	10.5 mm
Stimulus‐to‐LV activation time (stim‐LVAT)	72 ms
Paced QRS morphology	RBBB (rsR′ in V1, qR in V6)
Selective capture threshold	0.8 V at 0.5 ms
Nonselective‐to‐selective transition	Observed

*Note:* All threshold measurements were performed at a pulse width of 0.5 ms.

Abbreviations: LBBAP, left bundle branch area pacing; LV, left ventricular; RBBB, right bundle branch block; stim‐LVAT, stimulus‐to‐left ventricular activation time.

## 4 Conclusion and Results

### 4.1 Outcome and Follow‐Up

The patient was not prescribed any heart failure medication (ACEi, ARB, beta‐blocker, MRA, SGLT2i, GLP‐1RA, or vericiguat) before or after the procedure. At 2 months, dyspnea had resolved, NT‐proBNP was 90 pg/mL, LVEDD reduced to 50 mm, and LVEF improved to 45% (Figure 3C–E). At 6 months, LVEF was 51% and LVEDD 53 mm. At 1 year, LVEF reached 52%, LVEDD 48 mm, LVEDV of 108 mL, and LVESV of 47 mL. Device interrogation confirmed ventricular pacing burden remained 99%–100% (LBBAP) at all follow‐ups. No device‐related complications occurred. Pacemaker programming parameters were optimal (Table 1).

### 4.2 Conclusion

This case demonstrates the feasibility and potential benefit of upgrading to LBBAP for PICM, with sustained clinical and echocardiographic improvement. LBBAP may represent a promising physiological alternative to BiVP in selected patients, but larger comparative studies are needed to confirm efficacy and safety.

## 5 Discussion

Right ventricular pacing (RVP) can induce PICM in 20%–30% of patients. The conventional upgrade to BiVP has well‐documented benefits [1], but LBBAP has emerged as a physiological alternative. This single case does not intend to claim superiority of LBBAP over BiVP; rather, it highlights four specific insights that add to the existing literature.

First, successful LBBAP despite an intraventricular septal occluder. The patient underwent ventricular septal defect closure with an occluder device 14 years earlier. Septal hardware theoretically increases risks of lead entrapment, perforation, or unstable fixation. To our knowledge, this is among few reports demonstrating that LBBAP can be safely performed with an occluder in situ. The lead was advanced to a septal depth of 10.5 mm without complication, suggesting that LBBAP remains feasible even in complex septal anatomy.

Second, remarkably rapid reverse remodeling without heart failure medication. Within 2 months of LBBAP upgrade, LVEF improved from 35% to 45%, LVEDD decreased from 65 to 50 mm, and NT‐proBNP normalized. At 1 year, LVEF reached 52% and LVEDD 48 mm. Notably, the patient received no ACE inhibitors, beta‐blockers, or other heart failure drugs before or after the procedure. This absence of pharmacological confounders strongly attributes the recovery to LBBAP itself. The speed and completeness of reverse remodeling contrast with the slower, sometimes incomplete response observed with BiVP upgrades, particularly in patients with long‐standing dyssynchrony [2].

Third, sustained benefit at one‐year follow‐up. While short‐term hemodynamic improvements after LBBAP have been reported, long‐term data remain limited. This case documents sustained echocardiographic and functional improvement at 12 months, with stable pacing parameters and no device‐related complications, supporting the durability of LBBAP for PICM.

Fourth, contralateral implant overcoming venous obstruction. The original right‐sided leads could not be revised due to subclavian vein stenosis and adhesions from prior infection. The successful left‐sided LBBAP implant (new atrial lead + LBBAP lead, generator repositioned) provides a practical strategy for managing challenging lead revisions without requiring complex venoplasty or lead extraction.

Why LBBAP over conventional BiVP in this specific patient? Several patient‐specific factors guided this choice: (1) the presence of a septal occluder might theoretically impede coronary sinus lead placement (due to peridevice fibrosis or distortion of the ostium) or increase perforation risk (due to venous adherence or septal scarring); (2) prior pocket infection and subclavian stenosis favored a single‐lead approach to reduce re‐infection risk and procedure complexity; (3) the existing non‐MRI‐compatible generator could be repurposed with LBBAP, avoiding an additional coronary sinus lead; and (4) patient preference after shared decision‐making. These considerations are case‐specific and do not imply that LBBAP should routinely replace BiVP.

### 5.1 Potential Mechanisms of Reverse Remodeling

At the cellular level, LBBAP restores synchronous ventricular contraction, reducing mechanical dyssynchrony and wall stress. A recent study investigating epigenetic mechanisms found that patients who responded to LBBAP exhibited significant modulation of specific microRNAs (miR‐26, miR‐29, miR‐30, miR‐92, and miR‐145). Among these, the increase in miR‐30 levels correlated positively with improvements in LVEF and exercise capacity, suggesting that epigenetic regulation via microRNAs may contribute to the clinical benefits of LBBAP [5].

Although the patient received no heart failure medications, it is noteworthy that contemporary pharmacotherapies—including SGLT2 inhibitors, GLP‐1 receptor agonists, and vericiguat—have been shown to exert anti‐remodeling effects and improve LVEF in heart failure populations, including some CRT non‐responders [6, 7, 8, 9, 10]. Their absence in this case, however, eliminates potential confounding and allows the observed reverse remodeling to be attributed solely to LBBAP.

### 5.2 Limitations

Cardiac magnetic resonance was contraindicated due to a non‐MRI‐compatible device; thus, myocardial scar or subtle infiltration cannot be fully excluded, though the dramatic recovery makes alternative etiologies unlikely. Global longitudinal strain data were not available. As a single case report, the findings are hypothesis‐generating and require confirmation in larger comparative studies. For now, BiVP remains the established standard for CRT candidates, and LBBAP should be considered as an alternative in selected patients with specific anatomical or clinical constraints.

## Author Contributions

**Mengqi Yeh:** conceptualization, data curation, investigation, writing – original draft. **Shunsong Chen:** data curation. **Shanshan He:** data curation, writing – review and editing. **Ke Liu:** visualization, writing – review and editing. **Kai Yang:** data curation, writing – review and editing. **Jiang Wang:** data curation. **Zhiying Zhao:** data curation. **Feiyan Shang:** data curation. **Jinrui Guo:** conceptualization, project administration, supervision, writing – review and editing.

## Funding

The authors have nothing to report.

## Consent

Written informed consent was obtained from the patient to publish this report in accordance with the journal's patient consent policy.

## Conflicts of Interest

The authors declare no conflicts of interest.

## Data Availability

The data that support the findings of this study are available from the corresponding author upon reasonable request.
